# Supplementary material for: Evidence of torpor in the tusks of Lystrosaurus from the Early Triassic of Antarctica
Source: Commun Biol. 2020 Aug 27;3:471. doi: 10.1038/s42003-020-01207-6 (PMC7453012; doi:10.1038/s42003-020-01207-6)
Supplement: Supplementary file 1 — Supplementary Information [file 42003_2020_1207_MOESM1_ESM.pdf]

SUPPLEMENTARY FIGURES

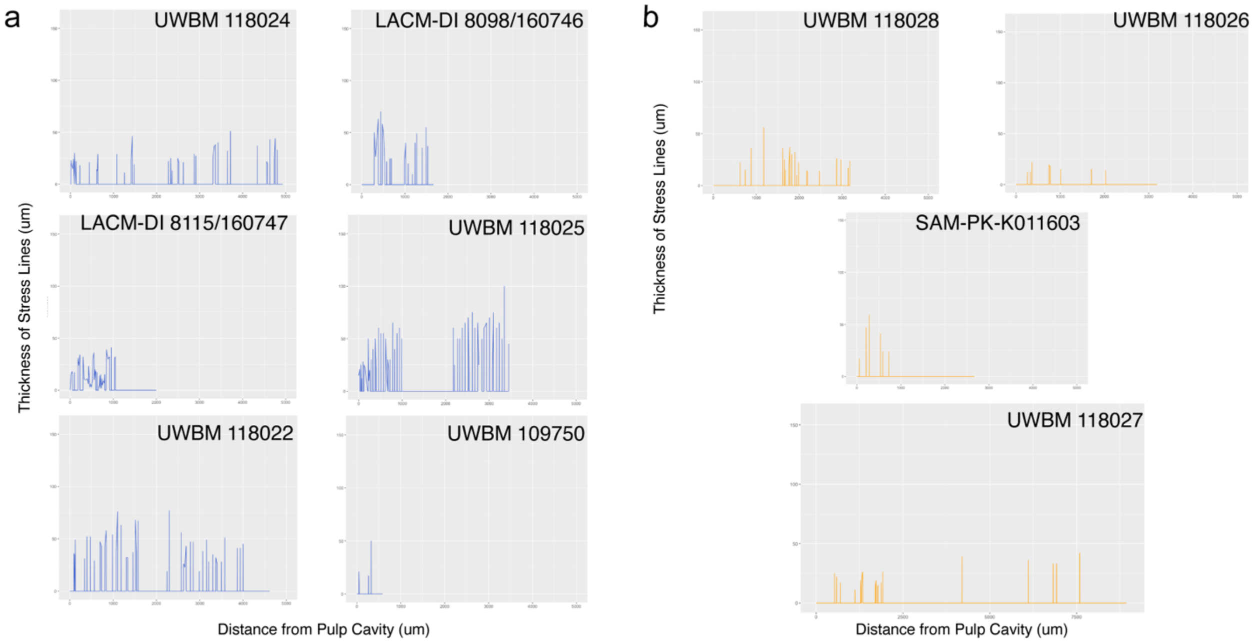

Supplementary Figure 1. Entire transect data on stress lines for both Antarctic (A) and South African (B) specimens to visualize patterns of stress observed along the tusk.

SUPPLEMENTARY TABLES

Supplementary Data Table 1. Specimen locality information.

Specimen Number	Inferred Paleolatitude	Locality Description
UWBM 118022	72° S	Coalsack Bluff in the Beardmore Glacier region of the Transantarcitc Mountains within 30 meters of the base of the Fremouw Formation
UWBM 118024	72° S	
UWBM 118025	72° S	
UWBM 109750	72° S	
LACM-DI 8115/160747	73° S	Collinson Ridge in the Shackleton Glacier region of the Transantarctic Mountains within 80 meters of the base of the Fremouw Formation
LACM-DI 8098/160746	71° S	McIntosh Ridge in the Shackleton Glacier region of the Transantarctic Mountains
UWBM 118026	61° S	approximately 3 meters below the Permo-Triassic boundary in the Bethulie District, Eastern Cape Province of South Africa
UWBM 118027	58° S	7 meters above the Permo-Triassic Boundary in the Namakwa District, Northern Cape Province of South Africa
UWBM 118028	62° S	5 meters above a major sandstone package of the basal Katberg Formation in the Nieu Bethesda District, Eastern Cape Province of South Africa
SAM-PK-K011603	62° S	approximately 22 meters above the boundary from the Nieu Bethesda District, Eastern Cape Province of South Africa

Antarctic specimens (above double line) and South African specimens (below double line) with inferred paleolatitude. Institutional abbreviations: **LACM**, Natural History Museum of Los Angeles County, Los Angeles, California; **SAM**, Iziko: South African Museum, Cape Town, South Africa; **UWBM**, University of Washington Burke Museum, Seattle, Washington.
