# Supplementary material for: Evidence of torpor in the tusks of Lystrosaurus from the Early Triassic of Antarctica
Source: Commun Biol. 2020 Aug 27;3:471. doi: 10.1038/s42003-020-01207-6 (PMC7453012; doi:10.1038/s42003-020-01207-6)
Supplement: Supplementary file 2 — Description of Additional Supplementary Files [file 42003_2020_1207_MOESM2_ESM.pdf]

Description of Additional Supplementary Files

File Name: **Supplementary Data 1**

Description: Our source data has been uploaded as Supplementary Data 1. It includes an Excel Workbook with four tabs with specimen numbers and locality (SA = South Africa; ANT or A = Antarctica). 'Specimen Averages' provides means for each specimen; 'Individual Growth Mark Data' provides individual measurements for line thickness, the distance between consecutive stress lines, as well as the distance between consecutive regular growth marks; the transect data used to make Fig. 3 and Supplementary Fig. 1 (previously Supplementary Fig. 1 G and H) is separated by locality to give two sheets, 'ANT Stress Mark Transect Data' and 'SA Stress Mark Transect Data'.
