# Supplementary material for: Evidence of torpor in the tusks of Lystrosaurus from the Early Triassic of Antarctica
Source: Commun Biol. 2020 Aug 27;3:471. doi: 10.1038/s42003-020-01207-6 (PMC7453012; doi:10.1038/s42003-020-01207-6)
Supplement: Supplementary file 4 — Reporting Summary [file 42003_2020_1207_MOESM4_ESM.pdf]

nature research

Corresponding author(s): Megan Whitney

Last updated by author(s): 6/9/2020

Reporting Summary

Nature Research wishes to improve the reproducibility of the work that we publish. This form provides structure for consistency and transparency in reporting. For further information on Nature Research policies, see our Editorial Policies and the Editorial Policy Checklist.

Statistics

For all statistical analyses, confirm that the following items are present in the figure legend, table legend, main text, or Methods section.

n/a	Confirmed	
☐	☒	The exact sample size (n) for each experimental group/condition, given as a discrete number and unit of measurement
☒	☐	A statement on whether measurements were taken from distinct samples or whether the same sample was measured repeatedly
☐	☒	The statistical test(s) used AND whether they are one- or two-sided<br>*Only common tests should be described solely by name; describe more complex techniques in the Methods section.*
☒	☐	A description of all covariates tested
☒	☐	A description of any assumptions or corrections, such as tests of normality and adjustment for multiple comparisons
☒	☐	A full description of the statistical parameters including central tendency (e.g. means) or other basic estimates (e.g. regression coefficient) AND variation (e.g. standard deviation) or associated estimates of uncertainty (e.g. confidence intervals)
☐	☒	For null hypothesis testing, the test statistic (e.g. F, t, r) with confidence intervals, effect sizes, degrees of freedom and P value noted<br>*Give P values as exact values whenever suitable.*
☒	☐	For Bayesian analysis, information on the choice of priors and Markov chain Monte Carlo settings
☒	☐	For hierarchical and complex designs, identification of the appropriate level for tests and full reporting of outcomes
☒	☐	Estimates of effect sizes (e.g. Cohen's d, Pearson's r), indicating how they were calculated

Our web collection on statistics for biologists contains articles on many of the points above.

Software and code

Policy information about availability of computer code

Data collection	Nikon NIS Elements
Data analysis	R

For manuscripts utilizing custom algorithms or software that are central to the research but not yet described in published literature, software must be made available to editors and reviewers. We strongly encourage code deposition in a community repository (e.g. GitHub). See the Nature Research guidelines for submitting code & software for further information.

Data

Policy information about availability of data

All manuscripts must include a data availability statement. This statement should provide the following information, where applicable:

- Accession codes, unique identifiers, or web links for publicly available datasets
- A list of figures that have associated raw data
- A description of any restrictions on data availability

Histological images that support this study have been deposited in MorphoBank with the project number 3650 at http://morphobank.org/permalink/?P3650.

Field-specific reporting

Please select the one below that is the best fit for your research. If you are not sure, read the appropriate sections before making your selection.

☐ Life sciences ☐ Behavioural & social sciences ☒ Ecological, evolutionary & environmental sciences

For a reference copy of the document with all sections, see nature.com/documents/nr-reporting-summary-flat.pdf

Ecological, evolutionary & environmental sciences study design

All studies must disclose on these points even when the disclosure is negative.

Study description	Fossils from Antarctic localities were compared to those from South African localities.
Research sample	Thin sections of six Antarctic tusks and four South African tusks were made available for this study.
Sampling strategy	Antarctic tusks were made from museum collections and fieldwork. All tusks that were available for sectioning were included. South African tusks were thin sectioned for previous research projects and novel data was collected from these existing thin-sections.
Data collection	Data was collected using NIS Elements light intensity readings that indicate relative dips and peaks in light. These changes in light were confirmed as regular or stress marks. The distance and thickness between stress marks as well as the distance between regular growth marks were recorded by M. Whitney
Timing and spatial scale	These data were collected from 2017-2019 as new fieldwork specimens were made available.
Data exclusions	No data were excluded
Reproducibility	Most specimens were examined more than once to confirm results.
Randomization	The nature of the fossil record limits randomization.
Blinding	During data collection, specimen number and locality were left blind to collector.

Did the study involve field work? ☒ Yes ☐ No

Field work, collection and transport

Field conditions	Conditions did not impact the fossil material.
Location	Shackleton Glacier Field Camp, Antarctica
Access & import/export	All permitting was conducted with the US Antarctic Program.
Disturbance	Field team followed Antarctic Treaty guidelines to minimize disturbance.

Reporting for specific materials, systems and methods

We require information from authors about some types of materials, experimental systems and methods used in many studies. Here, indicate whether each material, system or method listed is relevant to your study. If you are not sure if a list item applies to your research, read the appropriate section before selecting a response.

Materials & experimental systems

n/a	Involved in the study	
☒	☐	Antibodies
☒	☐	Eukaryotic cell lines
☐	☒	Palaeontology and archaeology
☒	☐	Animals and other organisms
☒	☐	Human research participants
☒	☐	Clinical data
☒	☐	Dual use research of concern

Methods

n/a	Involved in the study	
☒	☐	ChIP-seq
☒	☐	Flow cytometry
☒	☐	MRI-based neuroimaging

Palaeontology and Archaeology

Specimen provenance	Antarctic specimens were collected from the Shackleton Glacier region of the Transantarctic Mountains. All collections and exporting were made with the permission and support of the US Antarctic Program (PLR-1341304). South African specimens included previously made thin-section of collections material from the Karoo Basin of South Africa and depostied at the Iziko South African Museum.
Specimen deposition	Specimens (including thin-sections) are housed at University of Burke Museum, Natural History Museum LA County, and Iziko South African Museum.
Dating methods	No new dates provided

☐ Tick this box to confirm that the raw and calibrated dates are available in the paper or in Supplementary Information.

Ethics oversight	No ethical approval or guidelines were required since specimens were collected either from previously curated and accredited repositories or collected in accordance with the International Antarctic Treaty.

Note that full information on the approval of the study protocol must also be provided in the manuscript.
